# Socio-demographic transformations and living conditions among two indigenous and black populations in Northern Cauca during the period of 1993-2005

**Published:** 2012-06-30

**Authors:** Fernando Urrea Giraldo, Diego Alejandro Rodríguez Sánchez

**Affiliations:** a Sociologist, Full Professor, Department of Social Sciences, School of Social Sciences and Economics, Universidad del Valle, Cali, Colombia.; b Economist and sociology student in the Department of Social Sciences, School of Social Sciences and Economics, Universidad del Valle.

**Keywords:** Population characteristics, fertility, mortality, sociodemographic characteristics, living conditions, ethnic-racial groups

## Abstract

**Objectives:**

To describe the changes that occurred in some patterns of socio-demographic variables and in living conditions among the Nasa, Guambiana and Afrocolombian populations in the northern region of the Department of Cauca, and those occurring in two residential communities, one white-mestizo and one black, in Cali during the 1993-2005 period.

**Methods:**

This paper presents a descriptive study that analyzes several socio-demographic indicators from the census of 1993 and 2005, the specific data include: rate of juvenile dependency; total masculinity index; average size of the household; specific global and local birth rates, and infant mortality rates; life expectancy at birth; average years of schooling; health cover age status; and percentage of the population with unmet basic needs (UBN). In this way, it is possible to note differences in the course of socio-demographic evolution and in the standard of living trends in the differing populations under study.

**Results:**

The Guambiana Indian population in the municipality of Silvia presents lower birth rates than the Nasa population, characterized by their seasonal birth rates. Differing from the pattern of the indigenous people of Northern Cauca, the Afro-Colombian population both from this region and from the population residing in the urban zones of Cali's tend to show similar socio-demographic patterns.

**Conclusions:**

Although there have been profound changes recorded during this period among these populations under study, the ethnic-racial inequalities and those of social class seem to persist. From this first diagnosis, attention is called to the need for a more adequate reproductive health policy to attend the specific needs presented by the indigenous population.

## Abstract (es)

**Objetivos:**

Describir las transformaciones ocurridas en los patrones sociodemográficos y en las condiciones de vida de las poblaciones Nasa, Guambiana y afrocolombiana en el norte del departamento del Cauca, y la de dos sectores residenciales en Cali, uno blanco-mestizo y otro negro, durante el período 1993-2005.

**Métodos:**

Se trata de un estudio descriptivo a partir de los censos de población de 1993 y 2005, que analiza varios indicadores socio demográficos, procesando los microdatos censales: tasa de dependencia juvenil; índice total de masculinidad; tamaño promedio del hogar; tasas de fecundidad global y específicas, y de mortalidad infantil; esperanza de vida al nacer; promedio de años de estudio; cobertura en salud; y porcentaje de población con necesidades básicas insatisfechas. Así, es posible encontrar la existencia de diferencias notorias en la evolución sociodemográfica y en las condiciones de vida de las distintas poblaciones bajo análisis.

**Resultados:**

La población Guambiana del municipio de Silvia presenta tasas de fecundidad menores en comparación con la población Nasa, caracterizada por presentar tasas de fecundidad temprana. A diferencia de los pueblos indígenas norte caucanos, la población afrocolombiana de esta región y la residente en la zona urbana de Cali de clases populares negras tienden a presentar similares patrones sociodemográficos.

**Conclusiones:**

No obstante las profundas transformaciones registradas en este período entre estas poblaciones, las desigualdades étnico-raciales y de clase social parecieran persistir. A partir de este primer diagnóstico, se advierte la necesidad de una política de salud reproductiva más adecuada para las necesidades específicas que presenta la población indígena.

## Introduction

This article aims to analyze the socio-demographic changes and changes in the living conditions during the period from 1993 to 2005. Included in the analysis are the Nasa population, the Guambiano population (Misak) and the Northern Cauca Black or Afro-Colombian population that start from a series of special processing of micro-data from both censuses conducted by the Center for Socioeconomic Research and Documentation (CIDSE) at the Universidad del Valle, in collaboration with the National Administrative Department of Statistics (DANE). 

The study focuses on some key reservation areas - the municipalities of Caldono, Corinto, Jambaló, Piendamó, Toribio and Silvia- contrasting their situation with the two eastern municipalities of Cauca that are predominantly Nasa homelands, the regions of "Tierradentro" (Inzá and Paez) , and for the black population on the plains, encompassing the municipalities of Puerto Tejada and Villarrica. In turn, changes occurring in two urban clusters of Cali are analyzed, here referred to as the conglomerate Eastern Cali region (communities 7, 13 , 14, 15 and 21) and along the North-South corridor (communities 2, 17, 19 and 22) observing in both areas the changes in the black and white-mestizo populations. Thirdly, the aim is also to analyze the urban indigenous population (all Nasa and Guambiana populations) for the whole city of Cali.

By focusing on two urban conglomerates it is possible to approach the social class factor, assuming that it deals with two regions that bind the city: that of the East, the lower classes with the lowest living standards in the urban area, and theNorth-South corridor,more affluent middle classes and upper classes in Cali, so that in the analysis the factor of social class inter sects with the ethnic-racial^1^.

Among the indicators on which this descriptive analysis is based are: the ethnic-racial percentage of the population by municipality groupings and urban centers, the percentage of the population in the principal part of municipality, the rate of youth dependency, the overall masculine gender rate, the average household size, the total birth rate and the specific birth rates graphically presented, infant mortality rates (IMR), life expectancy at birth, and the average years of schooling for the population between 18 and 25 years, health insurance coverage (enrolment in the contributory and subsidized regimes taken together) and the percentage of population with at least one unmet basic need (UBN). The infant mortality rates and life expectancy were calculated by the authors based on survival data for live births by year of age on the two censuses, taking into account estimates from DANE for infant mortality and life expectancy from the censuses of 1985, 1993 and 2005^2^. The results for the departments, i.e. provinces or states, of Cauca and Valle de Cauca served as both a reference and that also took into account the classical studies on the indigenous population as a control population^3^ ^-^ ^4^.

For the calculation of the IMRthe indirect method of Trussell^5^ and Sullivan^6^ were used as a base, of children-as-survivors that are reported in census data, and Coale-Trussell variant exemplified through the study of Stover and Kirmeyer^7^ ^-^ ^8^. The data for clusters in Cali were from the 1993 census, by ethnic group. These were supported by the legal appeals relied on by the CIDSE-IRD^9^ from the microdata of this census by communities, which allowed for estimating the population of heads of household or spouses who were born in a municipality with historic Afro Colombia majorities, and comparing it to other municipalities with origins different from Cali. This facilitated having a first approximation of the two populations (Afro-Colombian origin and white-mestizos of non-Afro-Colombian origin), although it remained outside the Afro Colombian population that were born in Cali. However, through the results from the CIDSE-IRD survey of Afro-Colombians in Cali for 1998, which is representative for the entire black population in the city, migrants and natives of that year, we proceeded to adjust estimates of black and non black population from the census of 1993 for the two clusters.

**Figure 1:**
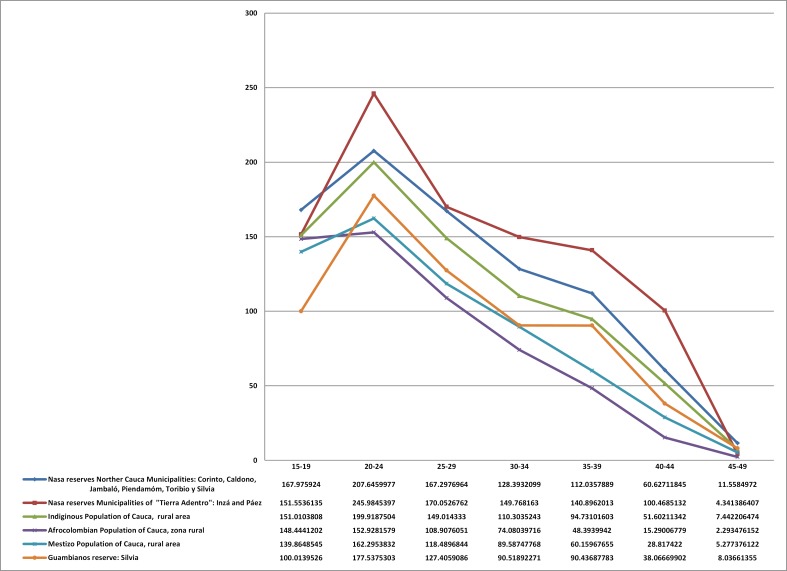
Specific fertility rates and Guambiano Nasa guards, for some municipalities and rural Department of Cauca by ethnic group 2005

Many of the indicators are disaggregated by sex. Upon proceeding to the analysis of specific birth rates also introduced for the indigenous populations on the Nasa and Guambiano reservation areas other comparative ethnic groups on the same territorial scale of Cauca, as seen in Figure 1 (all of the indigenous, Afro-Colombian and mestizo populations of the department in its rural areas). This allows greater clarity with the observations of changes that occurred between the two censuses in Nasa and Guambiana populations in reservation locations and allowed for comparisons with those found for indigenous people living in Cali. Thus, and as recorded in the results, the analysis could be relevant for the health sector as the reproductive components, attention to children in the population (e.g., less than 1 year of age but extending up to 5 years), and health coverage in different ethnic populations that vary according to ethnic-racial group, social class and urbanization.

The analysis describes the interaction of socio-demographic structural variables, such as those related to dependency, fertility, mortality and life expectancy at birth and those that touched on educational levels, and a summary indicator of living conditions such as that of the NBI. In this context, these first empirical results based on census information could be highly use full for planning health policies geared toward determined population group according to social class, ethnic-racial status and location.

In the municipalities of Buenos Aires, Cajibío, Caldono, Caloto, Corinto, El Tambo, Inzá, Jambaló, Miranda, Morales, Padilla, Paez, Piendamó, Puerto Tejada, Santander de Quilichao, Silvia, Suárez, Toribío, Totoró, Villarica and Guachené for 1993, of the total population, 7.3% were classified as Afro-Colombians and 25.3% as Indigenous, which is more than one third of its total population. However, it is very likely that there was major under-reporting in the 1993 census for these populations,which may even be more than 50.0% of the total. In the rural areas of Cauca, in which 42% of the population lived in 1993, one third of its population was listed as indigenous and 9.0% as Afro-Colombians. In 2005 21 municipalities were considered for the study with 29.6% of the population of the department self-reporting as Black or Afro-Colombian, and 34.1% as indigenous under differing population identities; i.e. 63.7% of North and Eastern Cauca constitute an Indian-Black region (in rural areas, this percentage is as high as 66.1% for the 21 municipalities)^10^.

A second sociological determinant factor to consider is the urbanization process that crosses this whole region under the dynamic of extending the metropolitan area of Cali to the whole of northern Cauca in terms of a complex and diversified market for work in the agro-industrial sector and industrial investments by means of the Páez Act. Metropolitanization that was already apparent from the decade of the eighties is related to the labor dynamics of the region and is characterized by increases in the demand for domestics and construction workers. Along with this dynamic, there was also greater demand for high schools and colleges. The Páez Act through accelerating and intensifying the process deepened the structural transformation of labor in the Northern Cauca region. This is especially seen in the geography of the flatland areas of Cauca river valley. This process generates an effect of employment and educational attraction for mountainous populations on both sides, the Western and the Eastern, and for a large majority of indigenous people, including the Nasa and Guambiana people, along with other groups -for example, the Yanacona, Pastos, Inga and others^11^.

### A panorama of notable demographic changes in the period 1993-2005

Although in the Northern Cauca region studied is mainly composed of indigenous and black persons (Table 1), there has been a slight percentage of reduction between the two censuses due to the gradually increasing presence of white-mestizo immigrants in the principal part and in the adjacent rural areas annexed to the urban area, and in some municipalities more than others; for example, it is occurring in Silvia in the form of recreational farms. This presence has until now not presented a threat to indigenous community order or to the considerable weight that the black population carries in the municipalities analyzed. The principal parts of the Nasa municipalities are least developed, in particular regarding Tierradentro, which shows low growth or relative decline in their percentage share of the population. This shows a process of migration to larger urban centers and without such a flow being offset by the arrival of migrants from rural areas. People clearly prefer to migrate to larger urban centers and dynamic economies such as Cali, Popayan or Santander de Quilichao.

With regard to the municipalities of PuertoTejada and Villarrica, it is very likely that the two population censuses, as an effec to industrialization via the Paez Law, generated a demand for both male and female manual labor and "rural" male labor in these municipalities which are urbanizing rural areas of the municipalities by labor that work in factories and reside on country farms^12^.

Youth dependency rates show the expected changes. In all ethnic groups, including the black, white-mestizo and indigenous populations of Cali, there is a process of restructuring gage according to the dynamics of demographic transition in the four Northern Cauca areas and in the two urban conglomerates studied.

The middle classes and upper classes of Cali, especially for white-mestizos, show lower rates of juvenile dependency and at the same time a greater old age dependency. This does not represent, however, a high rate of total dependence for these classes given the sharp decline of young people in these groups. To the contrary, for the lower classes, especially in the black population in the eastern conglomerate of Cali, the largest urban youth units are presented. This in turn helps to explain the higher rates of total dependence present there (as indeed for 1993, although not included in the summary table, old age dependency rates are significantly lower among the Eastern Afro-Colombian conglomerate of Cali than among the white-mestizo; additionally, the total dependency ratio for the urban Afro-Colombian population is 0.72, while for the white-mestizo population it is 0.69; also for this same conglomerate in 2005, total dependency rates are for the Afro-Colombian population is 0.63, and for the white-mestizo it is 0.58).

Comparing the three areas of reservation municipalities for the Nasa and Guambiano with the two urban clusters and the black population of Puerto Tejada and Villarrica it was found that he Nasa seem to have the greatest demographic gap during the study period, despite the decline in juvenile dependency. This is particularly notable in the municipalities of Inzá and Paez, but also in the mountainous Nasa areas (e.g. Caldonó, Corinto, Jambaló, Piendamó, Silvia and Toribio), yet by 2005 they still had high rates of you the dependency (Table 1). In the Guambíano reservation areas since 1993 there is a population age structure with lower age gaps than exist in the Nasa areas. This profile deepened in 2005, which suggests that the protected areas have been showing changes in population structure for at least the past two decades.

**Table 1:**
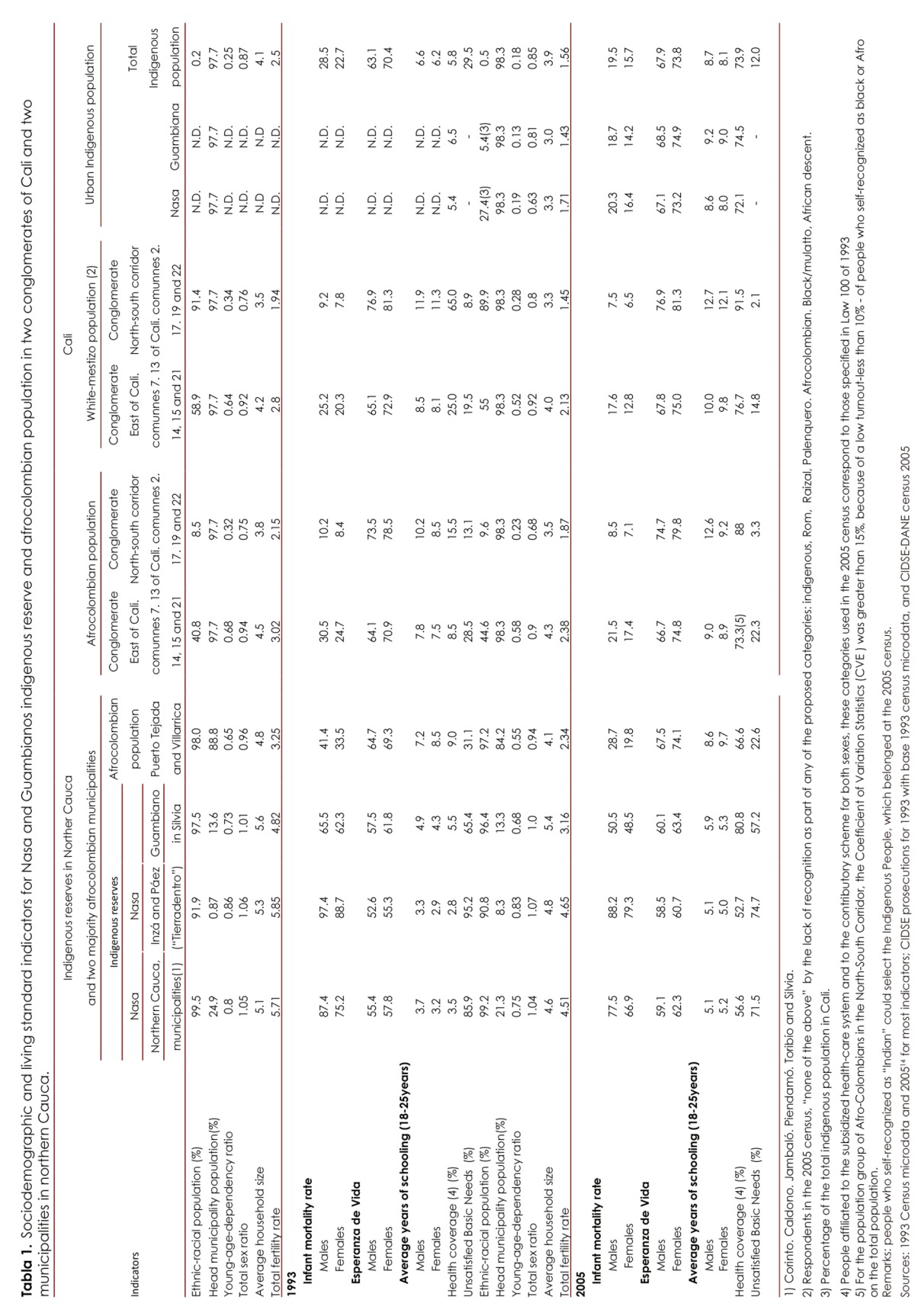
Sociodemographic and living standards and indicators for Nasa Guambinos guards, black population in two clusters of quality and northern Cauca.

In the rural reservation areas ,the total masculinity index remained above 1.0 in both censuses, which is especially noticeable among the Nasa people (Table 1). This is mainly related to female migration, a phenomenon similar to that for the whole rural farming population.The average household size corresponds to the age structures. As the demographic transition progresses, with the relative aging of the population,a smaller average household size was shown, with the exception of Guambiana population in Silvia which showed a higher average household size over the two censuses in spite of having fewer young people than the NASA. This fact is explained by the historical land scar city in Silvia and by the practice of newly-formed couples residing for a period in the household of the husband's parents, two social phenomena that reinforce each other^13^.

The total fertility rate - interacting with the patterns of the age structure and dependencies - systematically decreased between the two censuses but did so unevenly for the different populations under study (Table 1). This is the most prominent factor that would affect the population structure. However, it is noteworthy that among the Guambiana population for the 1993 census and among the Nasa population for the 2005 census, the historical fertility rates are lower than the global fertility rates. This implies an apparent rise in the fertility rate for women under 24 (as shown in Figure 1 for the youngest age groups of the Nasa population).

In contrast, among Guambiana women with the 2005 census there has been a collapse in the rates of early fertility (Fig. 1). Specific rates continue to lower in all five-year age groups when compared with hose among the Nasa women in Northern Cauca and Tierradentro areas. Similarly, they are lower than for all women among the indigenous groups in Cauca.In addition, specific rates appear to be very similar to those of the mestizo and Afro-Colombian rural populations of Cauca, but, unlike them, their teenage fertility rates would be the lowest (Fig. 1).

**Figure 2:**
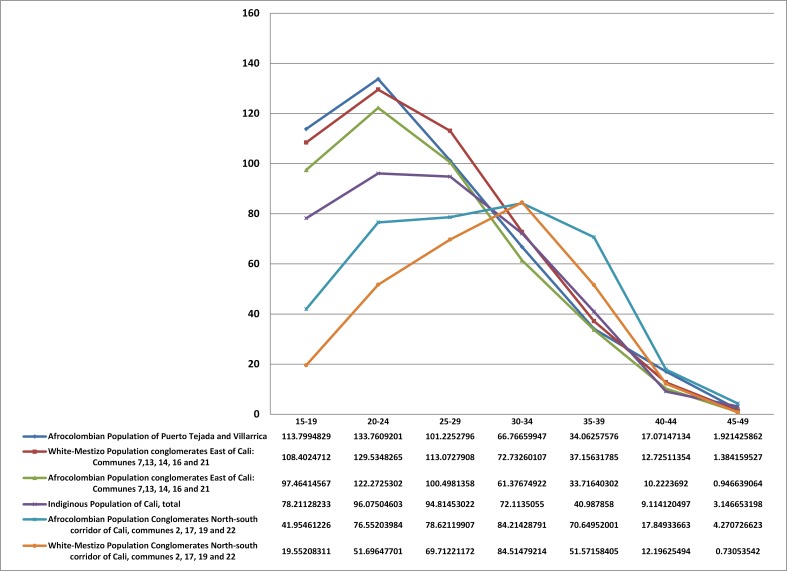
Specific fertility rates in two urban clusters in Cali and two municipalities of northern Cauca by ethnic group 2005

Among black women of Northern Cauca a significant reduction in fertility rates was observed between the two censuses (Table 1), and this reduction is similar to that observed in the 2005 census for black women in the East Cali urban communities. This is consistent with the pattern for black working class women in the greater metropolitan region to generally have available reproductive health services, and with specific fertility rates similar to other groups in this geographic cluster (Fig. 2).

It is worth noting that the specific fertility rates for the Cali conglomerate of middle classes and upper classes, white-mestizo and black sare not only the most moderate, but the reproductive peak occurs in the 30-34 age group. This is a pattern similar to that found in core capitalist countries with the highest human development index(Fig. 2).On the other hand, the indigenous population in Cali, which is high lighted by the Nasa and Guambiano groups, has a lower fertility rate than that of the mestizo and Afro-Colombian populations in the Eastern Cali conglomerate and in the municipalities of PuertoTejada and Villarrica (Fig. 2). This is consistent with the selective urban migration pattern of other indigenous groups, as has been supported by the findings of other recent studies.

### Changes in living conditions and socio-demographic variables: the role of education and increased health coverage.

The reduction in infant mortality rates between the two censuses for indigenous groups, the black population,and the white-mestizo population in Cali in the two clusters studied are consistent with the pattern of declining fertility rates in conditions of significant in equalities, according to ethnic-racial groupings, level of urbanization and social class. The reduction in infant mortality for both sexes and the total fertility rate is associated with the increases found in educational level indicators(increased literacy rate for both sexes in the age group of 15 years and older, and in the average years of schooling for the population between 18 to 25 years of age).

However, it is necessary to recall that the declines in infant mortality and fertility rates, as well as noted increases in educational levels also maintain significant inequalities by social class, ethnic-racial group and level of existing urban development. For example, the most rural populations of the Nasa reservation population in Tierradentro are associated with greater lags in the demographic transition process of their population structures.

Life expectancy at birth, correlated with the IMR, follows the same regional pattern of significant inequality with in the territorial areas studied. There is an axis for increasing life expectancy on approaching the urban epicenter and inside communities for those in the middle and upper classes, but at the same time this phenomenon crosses ethnic and racial lines. The indigenous Nasa people in their rural reservations constitute the group with the lowest life expectancy. Incidentally,urban indigenous people, although they have the least number of years of life expectancy in the city of Cali, when compared to groups in their native rural areas appear to have a better position (see Table 1). In reality, this group, which is part of the urban working classes, could be considered as relatively privileged in comparison with indigenous people in their rural areas of origin. 

The significant differential increase in health care coverage conforms to the socio-demographic trends described above (Table 1). However, this factor, along with the effect of education and the impact of the urbanization process, largely explains the significant reductions in the IMR and fertility rates found. It is also clearly seen in the considerable gap in health care coverage found in the protected rural areas of the Nasa people. On the other hand, there is less of a gap between the Guambiana populations from Silvia, and there are increasing gaps found among populations in the low land areas. Regardless, among the indigenous population with the lowest rates of health care coverage, suggests the hypothesis that the model of indigenous ARS would show a positive effect from the extension of the basic components in the health care system to these populations. A notable example is the Mama Dominga Hospital that serves the native Guambía people in the Silvia area, under the direction of indigenous authorities through the existing ARS model.

Finally, the differential in the UBN indicator reflects the substantial changes in the living conditions of the three reservation areas,the Northern Cauca Afro-Colombians, the indigenous city populations and the groups in the two urban clusters.There is a strong correspondence between this summary indicator and the evolution of the socio-demographic indicators previously discussed. The decline in poverty, measured by theUBN, while retaining the structural inequalities over time, has been quite systematically consistent.

## Conclusions

Changes in socio-demographic variables and living conditions observed for the two indigenous groups and the black populations in Northern Cauca in the period between1993 and 2005 when compared to populations located in the two urban conglomerates of Cali,including the case of the population of the urban indigenous population, revealed the existence of the following sociological phenomena:

1. Development of the demographic transition with a systematic reduction of overall fertility rates and the presence of modern urban behaviours among weal their classes are associated with higher educational levels for the female population and their entry into the labor market. This phenomenal so extends to the lower classes, but under strong limitations as to the levels of education possible and reduced health coverage. In this sense,the different ethnic and racial groups in the city would be most benefited but under conditions of inequality since the social sectors of the middle and upper classes benefit more in terms of life expectancy.

2. In the indigenous areas of the Nasa reservation the living conditions are the lowest, as evidenced by higher infant mortality rates; however,with the reported declines there is a general increase in life expectancy, although among those with lower average ages. By contrast, the Guambiana population stands out as an ethnic group while with in the dynamics of demographic transition are close to the behavioral patterns of the rural mestizo population and the urban working classes. This is associated with the incidence in this ethnic group of higher educational levels and broader health coverage. It is also the indigenous people with the highest educational levels, particularly for indigenous women, with respect to other indigenous groups living in Cali.

These conclusions allow the categorization of the results of this study as an input for restructuring reproductive health policies and those relating to maternal and child health for targeting different population groups according to ethnic-racial issues and factors of social class in accordance with urbanization patterns in the region. The region includes the broad metropolitan area to the south, the mountainous Northern Cauca area, but also the differing socials spheres in the very heart of the city; for example, those following the criteria of urban conglomerates. This restructuring should, however, be carried out with due respect shown for the autonomy of indigenous and black populations.

It is essential for epidemiological studies and clinical follow-up studies for the differing components of public health with respect to both indigenous population groups on reservations or in urban centers, but also to blacks, mestizos and whites, take into account the inter-sectionality of the socio-demographic variables and living conditions. This includes the field of health care coverage as a key factor as has been attempted through the description and analyses provided in this study.More than being a culturally sensitive look that distils ethnic populations and risks racism through arbitrarily stereotyping daily practices, we tried to determine the regulating social configurations in order to guide more appropriate and effective public health policies.
